# Evaluation of Bile Salts on the Survival and Modulation of Virulence of *Aliarcobacter butzleri*

**DOI:** 10.3390/antibiotics12091387

**Published:** 2023-08-30

**Authors:** Cristiana Mateus, Cláudio J. Maia, Fernanda Domingues, Roland Bücker, Mónica Oleastro, Susana Ferreira

**Affiliations:** 1 CICS-UBI—Health Sciences Research Centre, University of Beira Interior, 6200-506 Covilhã, Portugal; cristiana.lopes.mateus@ubi.pt (C.M.); cmaia@fcsaude.ubi.pt (C.J.M.); fcd@ubi.pt (F.D.); 2 Clinical Physiology/Nutritional Medicine, Medical Department of Gastroenterology, Infectiology, Rheumatology, Charité—Universitätsmedizin Berlin, 12203 Berlin, Germany; roland-felix.buecker@charite.de; 3 National Reference Laboratory for Gastrointestinal Infections, Department of Infectious Diseases, National Institute of Health Dr. Ricardo Jorge, Av. Padre Cruz, 1649-016 Lisbon, Portugal; monica.oleastro@insa.min-saude.pt

**Keywords:** *Aliarcobacter butzleri*, bile salts, virulence

## Abstract

*Aliarcobacter butzleri* is a Gram-negative bacterium associated with infections of the gastrointestinal tract and widely distributed in various environments. For successful infection, *A. butzleri* should be able to tolerate various stresses during gastrointestinal passage, such as bile. Bile represents an antimicrobial host barrier that acts against external noxious agents and consists of a variety of bile salts. The intestinal bile salts act as detergents involved in the antimicrobial host defense; although, on the bacterial side, they could also serve as a signal to activate virulence mechanisms. The aim of this work was to understand the effects of bile salts on the survival and virulence of *A. butzleri*. In our study, *A. butzleri* was able to survive in the presence of human physiological concentrations of bile salts. Regarding the virulence features, an increase in cellular hydrophobicity, a decrease in motility and expression of *flaA* gene, as well as an increase in biofilm formation with a concomitant change in the type of biofilm structure were observed in the presence of sub-inhibitory concentration of bile salts. Concerning adhesion and invasion ability, no significant difference was observed. Overall, the results demonstrated that *A. butzleri* is able to survive in physiological concentrations of bile salts and that exposure to bile salts could change its virulence mechanisms.

## 1. Introduction

*Aliarcobacter* genus belongs to the *Arcobacteraceae* family and the *Campylobacterales* order [1,2,3]. These bacteria are characterized as small Gram-negative, S-shaped or helical cells, motile by a single polar flagellum, and capable of growing at temperatures from 15 °C to 42 °C [1]. Among the species included in this genus, *A. butzleri*, *A. cryaerophilus*, *A. skirrowii*, *A. thereius* and *A. lanthieri* have been associated with human disease, such as enteritis and bacteremia, accompanied by various symptoms, for example diarrhea, abdominal cramps, nausea, fever and vomiting [1,4,5,6,7,8]. These species can be found in different hosts and environments, and can be isolated from different sources, such as water, food, animal and human samples [4,7,9,10]. Considering the environmental distribution, disease association and reported outbreaks, the consumption of food and water contaminated with *Aliarcobacter* spp. has been considered the main route of transmission of these bacteria to humans [4,7,9]. Among the species mentioned, *A. butzleri* has been highlighted as an emerging gastrointestinal pathogen, with worldwide distribution, and being considered the fourth most frequent *Campylobacter*-like organism found in human diarrheic samples, including patients with enteritis [6,7,11,12]. Furthermore, the ability of *A. butzleri* for adherence, invasion and intracellular survival in intestinal epithelial cells, in addition to cytotoxic effects supports its pathogenic potential [4,7,9,13,14].

Several factors affect the gut persistence ability of microorganisms, such as the gut microbiota that provides protection against infections and colonization resistance, through multiple mechanisms, including competition for nutrients, support of epithelial barrier integrity (mucus layers), bacteriophage implantation, immune activation, and secretion of antimicrobial products, like bacteriocins, short chain fatty acids and secondary bile acids [15]. Besides that, bacteria are exposed to several other adverse conditions. In fact, as an enteropathogen, *A. butzleri* is exposed to several adverse host factors during its passage through the gastrointestinal tract, such as flotations in pH, osmotic pressure and the presence of harmful substances during its passage through the gastrointestinal tract, such as reactive oxygen and bile [16,17]. The bile is produced by hepatocytes in the liver, concentrated and stored in the gallbladder, and subsequently released into the small intestine [16,17,18,19]. After digestion of lipids by lipases, the bile induces formation of micelles, which consists of long-chain fatty acids, glycerol and monoglycerides as well as apolar fatty acids, fat-soluble vitamins and the bile acids as emulsifier. The secreted bile in the small intestine contains a multitude of components, including, water, several bile salts, lipids (phospholipids, cholesterol, and fatty acids), proteins (globulins and albumins), ions, pigments, carbohydrates, vitamins and other trace elements [20,21,22]. Bile acts as a barrier against external noxious agents, which can provide host protection, acting as a detergent involved in host antimicrobial defense. Nonetheless, enteric pathogens resist the bactericidal activity of bile and are able to use it as one of the most intriguing host signals affecting virulence [16,20,23,24]. Amongst its constituents, the bile salts sodium cholate and sodium deoxycholate are described as virulence inducers of bacterial enteropathogens [16,20,24,25]. Bile salts are relevant physiological agents for intestinal absorption of nutrients and biliary secretion of lipids, toxic metabolites, and xenobiotics [22]. After release from the gallbladder, bile salts facilitate the emulsification of dietary fats and aid in the intestinal absorption of lipids and lipophilic vitamins, one of their main functions, being subsequently reabsorbed in the intestine and transported back to the liver [19,22]. The enterohepatic circulation of bile salts from the liver to the intestine and back to the liver is quite efficient, with approximately 95% of the bile salts reabsorbed [19]. This circulation thus plays a fundamental role in the absorption and distribution of nutrients, in metabolic regulation and in homeostasis [22].

Several mechanisms of resistance and virulence can be modulated in the presence of bile salts, to ensure that bacteria can survive, colonize and proliferate in the host [23,26]. In fact, bile salts were found to affect cellular adhesion and thus intestinal adhesion and biofilm formation. Biofilm is a common approach for bacterial pathogens to resist antimicrobial exposure, such as high levels of oxygen or bile [16,27], and many species are capable to enhance the formation of biofilm as a response to this stress [16,27,28]. Furthermore, bile salts, such as sodium deoxycholate, cholate and chenodeoxycholate, can modulate the adhesion and invasion of epithelial cells, in several bacterial pathogens, such as *Campylobacter jejuni*, *Escherichia coli*, *Shigella flexneri*, *Salmonella enterica* serovar Typhimurium, *Bacteroides fragilis* and *Vibrio parahaemolyticus* [27,29,30,31,32,33]. As adhesion to intestinal epithelial cells is required for establishment of infection, modulation of adhesion by bile may play an important role in successful colonization and infection. In fact, bile and bile salts present in the small intestine may act as a signal for bacteria to initiate colonization of the epithelium. Infection may be followed by tissue colonization and, in some cases, bacterial invasion of host cells, followed by intracellular proliferation, spread to other tissues, or persistence in the host [7,28].

In the presence of several unfavorable environmental factors, bacteria can change their behavior, through modulation of virulence mechanisms and protein expression, enabling responses to specific stresses. While studies have shown the potential of bile salts as a host signal for several enteric pathogens, the current knowledge regarding the response of *A. butzleri* to bile salts remains to be clarified. Considering that *A. butzleri* is an emerging pathogen, capable of infecting, colonizing and proliferating the human gastrointestinal tract [9,34], it is important investigate the response of this species to bile salts, by specifically evaluating the resistance to bile salts, as well as the role of this substrate as an environmental signal to regulate *A. butzleri* virulence.

## 2. Results

### 2.1. Effect of Bile Salts on the Growth, Aggregation and Motility of Aliarcobacter butzleri

**Figure 1:**
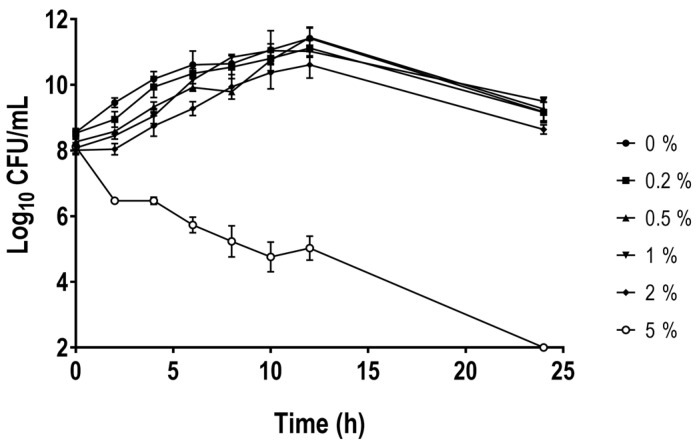
Effect of bile salts on the growth curve of *Aliarcobacter butzleri* AB_2811 by logarithmic counting CFU/mL in increasing concentrations of bile salts (0.2% to 5%). The graphic represents the mean ± SEM of at least three independent experiments.

To evaluate the effect of bile salts in the survival of *A. butzleri*, a range of physiological concentrations found in the gastrointestinal tract, from 0.2% to 2%, depending on circadian rhythm, diet and individual, and a concentration above this range (5%) were tested. The physiological range of concentrations of bile salts showed a low influence in *A. butzleri* Ab_2811 growth. However, the concentration above the physiological led to a reduction in survival to below the detection limit after 24 h of exposure (Figure 1). Thus, all subsequent analyses were performed with 0.2% of bile salts, a physiological concentration having no effect on bacterial growth when compared with growth in the absence of bile salts.

**Figure 2:**
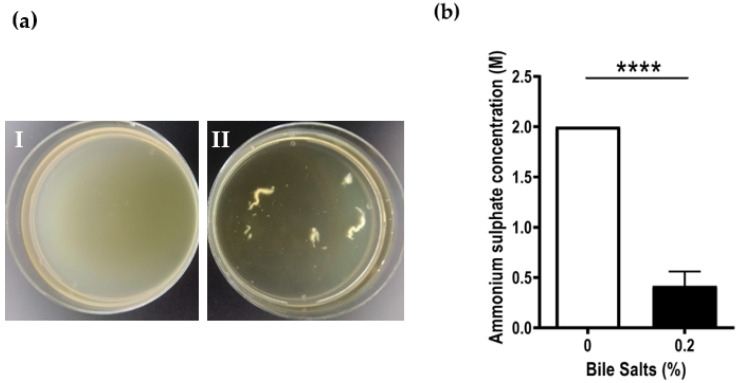
Effect of 0.2% of bile salts on cell hydrophobicity of *Aliarcobacter butzleri* Ab_2811. (**a**) Aggregation of *Aliarcobacter butzleri* Ab_2811 in absence (I) and presence (II) of 0.2% of bile salts (**b**) Cellular hydrophobicity by salting-out method. Each bar represents the mean ± SD of at least three independent experiments. **** *p* < 0.0001.

During the bacterial growth, formation of aggregates could be observed throughout the time of exposure to various concentrations of bile salts (Figure 2a); thus, the bacterial hydrophobicity was further evaluated using a salting-out method with ammonium sulphate. An increase in hydrophobicity of Ab_2811 in the presence of 0.2% of bile salts was observed, when compared to the bacteria incubated in the absence of the bile salts (Figure 2b). Considering this profile, prior to all subsequent assays the aggregates were previously dispersed by vortexing of the suspensions.

**Figure 3:**
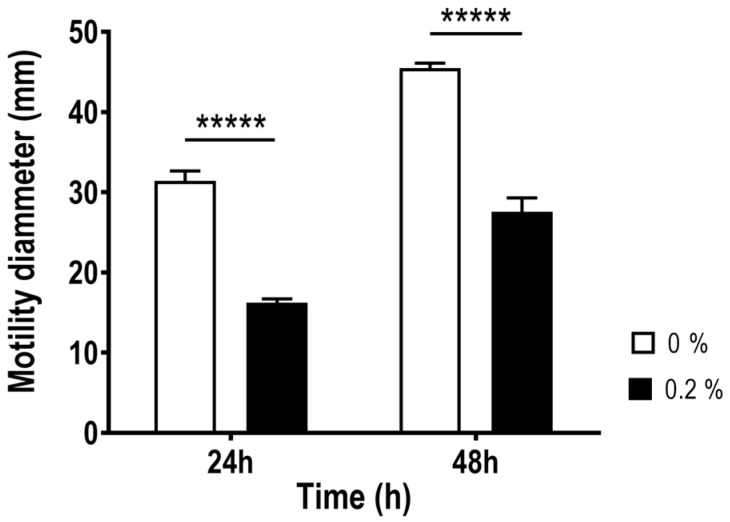
Bacterial motility of *Aliarcobacter butzleri* Ab_2811 in presence and absence of 0.2% of bile salts. Data match the mean ± SD of at least three independent experiments. ***** *p* < 0.00001.

Regarding the effect of bile salts on the motility of *A. butzleri* Ab_2811, a significant difference was observed at 24 and 48 h after the incubation with and without bile salts (Figure 3). These results showed that the bacterial motility of the strain decreases when exposed to bile salts.

### 2.2. Impact of Bile Salts on Biofilm Formation of Aliarcobacter butzleri

**Figure 4:**
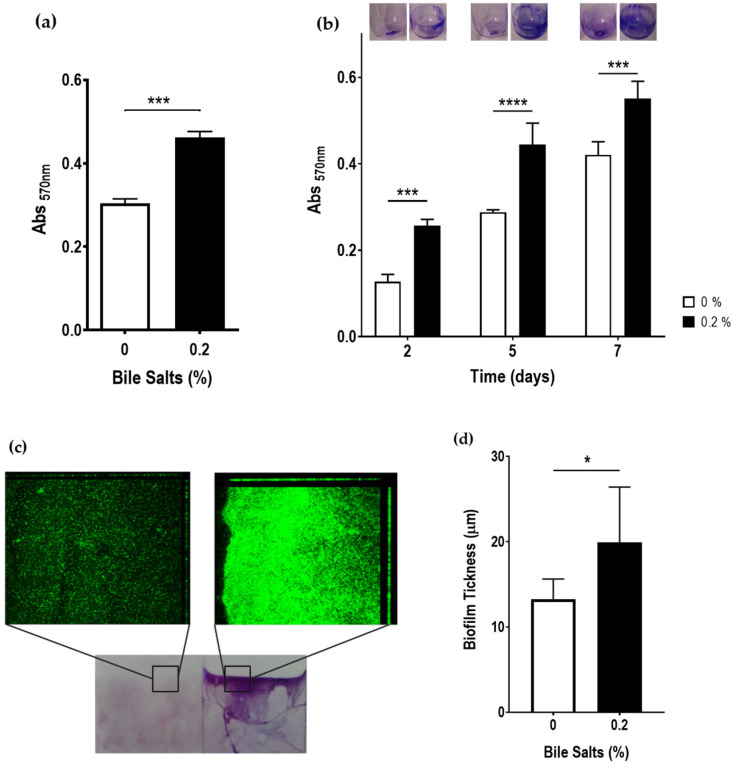
Evaluation of biofilm formation by violet crystal staining of *Aliarcobacter butzleri* AB_2811 grown in the presence and absence of 0.2% of bile salts (**a**) in 96-well U-bottom plates with 2 days of incubation and (**b**) glass tubes at three time points (2, 5 and 7 days). (**c**) 3D z-stacks of biofilm of *Aliarcobacter butzleri* Ab_2811 grown on glass coverslips stained with SYTO 9 in absence (left) and presence (right) of bile salts and (**d**) mean thicknesses of the biofilm analyzed using COMSTAT software. Each bar represents the mean ± SD of at least three independent experiments. * *p* < 0.05; *** *p* < 0.001; **** *p* < 0.0001.

The impact of bile salts on biofilm formation ability was assessed in 96-well plates and glass tubes (Figure 4). In the presence of bile salts, the biofilm-forming ability of the Ab_2811 strain significantly increased, in both experimental set-ups, when compared to the bacterial culture incubated without bile salts (Figure 4a). Additionally, an increased biofilm formation was measured after prolonged incubation time (Figure 4b). An incubation period of 5 days was chosen for the subsequent microscopy and evaluation of the biofilm thickness.

When Ab_2811 strain was exposed to bile salts, the formation of a pellicle in the interface air-medium was observed with both methods, microplate and glass tubes. Therefore, additional assays were performed to confirm and quantify this phenomenon. The phenotype change was confirmed by crystal violet staining of a coverslip vertically positioned during biofilm formation and the biofilm thickness corresponding to the pellicle was assessed. When observing the coverslip, a strong formation of biofilm at the interface air-medium was observed in the presence of bile salts, which was confirmed by confocal microscopy, where an increase in fluorescence was observed at the interface area in the presence of bile salts, when compared with the control (Figure 4c). An informatics analysis performed using COMSTAT software confirmed that, when considering the air-medium interface area, biofilm thickness was significantly higher in the presence of 0.2% of bile salts, when compared with the biofilm thickness in absence of bile salts (Figure 4d).

### 2.3. Effect of Bile Salts in the Expression of Five Virulence Genes of Aliarcobacter butzleri

**Figure 5:**
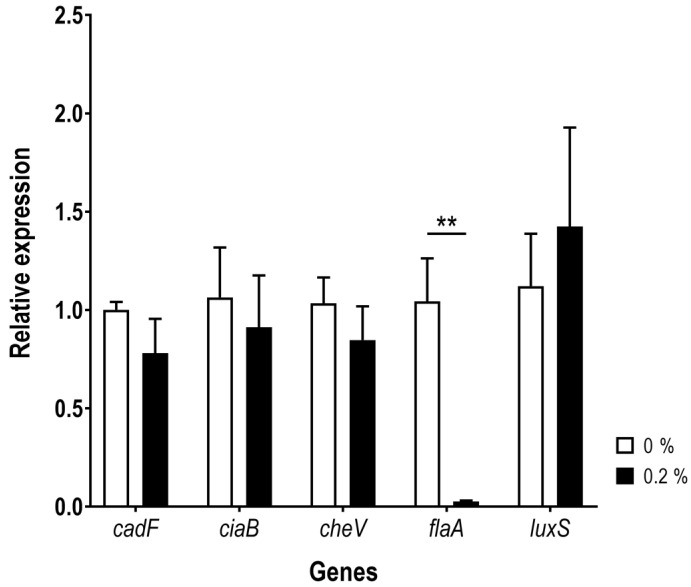
Relative expression of *cadF*, *ciaB*, *cheV*, *flaA* and *luxS* genes of *Aliarcobacter butzleri* Ab_2811 in the presence and absence of 0.2% of bile salts. Data correspond to the mean ± SEM of at least three independent experiments. ** *p* < 0.01.

We evaluated the relative expression of *cadF*, *ciaB*, *cheV*, *flaA*, and *luxS* genes of *A. butzleri* AB_2811 grown in presence or absence of bile salts by real-time PCR. A strong decrease in the expression of *flaA* gene was found, when the strain was exposed to 0.2% of bile salts, while an opposite trend was observed for *luxS* gene. On the other hand, no significant difference was observed for the *cadF*, *ciaB* and *cheV* genes in both tested conditions (Figure 5).

### 2.4. Effect of Bile Salts in the Adhesion and Invasion Abilities of Aliarcobacter butzleri to Caco-2 Cells

**Figure 6:**
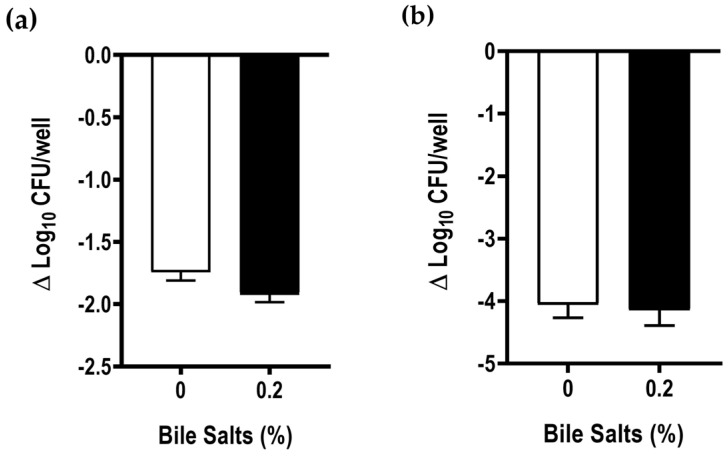
(**a**) Adhesion and (**b**) invasion of Caco-2 cell line, after 6 h exposure of *Aliarcobacter butzleri* Ab_2811 to 0.2% of bile salts. Bacterial survival was determined by variation in the CFU/mL logarithm after 3 h of incubation with the Caco-2 cell line. Data correspond the mean ± SEM of at least three independent experiments.

The capacity of *A. butzleri* to adhere and invade epithelial cells after exposure to bile salts was analyzed in Caco-2 cells. The results show that pre-exposure to 0.2% bile salts did not significantly influence the bacteria’s ability to adhere and invade the Caco-2 cell line (Figure 6).

## 3. Discussion

As an enteropathogen, *A. butzleri* must overcome numerous barriers to establish a successful infection, such as bile, which acts as harmful agent for bacteria outside the intestinal microbiota and could modulate several resistance and virulence mechanisms of the bacterium [4,7,16,17,23,35,36]. Several aspects of the virulence of this species are only now being elucidated, leaving a gap in our knowledge of how this bacterium might survive and migrate through the gastrointestinal tract. Thus, for the first time the virulence potential of *A. butzleri* considering its survival to bile salts, as well as the potential to modulate its virulence by these compounds was enlightened by this work.

Besides the digestive function of lipid absorption via micelles, bile salts are antibacterial compounds that may disrupt bacterial membranes, lead to DNA oxidative damage, denature proteins, or chelate iron and calcium [16,22,24]. Thus, firstly, the assessment of the planktonic growth of *A. butzleri*, in the absence and presence of physiological concentrations of bile salts and at higher concentration was performed. Although the bacterium was unable to survive at concentrations of 5% bile salts, it was able to survive at all concentrations tested in the range of physiological concentrations. This behavior has already been described for other microorganisms, such as *S. flexneri*, where a normal growth within the physiological range of bile salts was observed, but slowing and/or inhibition of growth occurred at concentrations above physiological levels [27]. Similarly, for *C. jejuni* and *E. coli* a decrease in bacterial growth can be associated with an increase in the concentration of bile or bile salt [28,37]. The presence of the bacterial wall provides a barrier to the absorption of different molecules; however, bile salts can enter the bacterial cell by diffusion or by passage through various existing porins, and consequently, some mechanisms are required to reduce its concentration within the cell, such as efflux pumps [16,24]. The presence of these efflux pumps in some bacteria can be correlated to the ability to survive in this condition. For example, the CmeABC multidrug efflux pump in *C. jejuni* or the AcrAB-TolC in *E. coli* and in *S. enterica* serovar Typhimurium have a clear role in bile salt resistance [38,39,40]. Previous work from our group showed that the use of the efflux pump inhibitor phenylalanine-arginine β-naphthylamide had influence on the resistance to sodium cholate, thus pointing to the role of efflux pumps in resistance to bile salts in *A. butzleri* [41]. The role of resistance-nodulation-cell division efflux pumps in bile salts resistance was also demonstrated by our group, where a knockdown of *areG* resulted in an 8-fold decrease in the minimum inhibitory concentration of the bile salt mix, highlighting the function of the AreGHI system in the *A. butzleri* response [42].

In the present work, following the growth analysis, a concentration of bile salts that did not affect bacterial growth was selected for the following assays (0.2%). The contact with bile salts led to the formation of aggregates, similarly to what was previously described for *S. flexneri*, *E. coli* and *B. fragilis* [27,28,30]. In fact, the observed aggregation correlated with a strong increase in the cellular hydrophobicity of the strain under study when previously exposed to 0.2% of bile salts, showing that bile salts play a role in altering the cellular hydrophobicity of *A. butzleri* Ab_2811. The formation of bacterial aggregates can be considered as a defense mechanism against biliary stress to which bacteria are subjected. Besides that, aggregation factors may also mediate binding to the host epithelium, and thus aid in other virulence mechanisms, such as biofilm development and, consequently, facilitate infection [28].

Besides the barrier role of bile salts, several enteric pathogens use these molecules as a signal to modulate virulence, regulating the colonization or maintenance of infection in the human gastrointestinal tract [16,19,23,24]. In fact, the resistance to bile, but also virulence modulation by bile may be critical to enteric pathogens’ success and survival [23]. Accordingly, considering bacterial motility, biofilm formation, host cell adhesion and invasion as mechanisms that play a role in the virulence of *A. butzleri* [4,7,9], these features were evaluated following exposure to bile salts. Motility is essential to bacteria’s ability to cause disease, influencing virulence mechanisms, such as biofilm formation, adhesion and invasion ability [7,9,43] that, consequently, modulating the pathogenicity of the bacterium. Regarding for *A. butzleri* motility, a strong decrease in the presence of 0.2% of bile salts was observed. This result is in line with data from literature for other enteropathogens, namely the significant reduction in motility in *E. coli* in the presence of glycol-conjugated cholate, a bile component [28], and in *Bacillus cereus*, where experiments in soft agar showed decreased motility in the presence of 0.005% bile salts [44]. In turn, the presence of bile acid deoxycholate had no effect in the bacterial motility of *C. jejuni* [25]. This decreased motility is likely associated with the decreased expression of *A. butzleri flaA* gene, similarly to what was described for *S. enterica* serovar Typhimurium that in response to the presence of bile showed a downregulation of several flagellar genes, such as *flhC*, *flgC* and *fliC* [45]. Different trends have been observed for other bacteria, such as *C. jejuni*, where an increased *flaA* gene expression was observed in the presence of bile salts [29,37,46]. As previously mentioned, motility is essential for host invasion, infection colonization and, for many bacteria, an important factor for biofilm formation [9,43]; indeed, in *C. jejuni* biofilm formation may be mediated through flagellar filament, and is induced by bile salts [47]. Induction of biofilm formation by bile and its components was also observed for several other enteric species, such as *Vibrio cholerae*, *B. fragilis*, *S. enterica* serovar Typhimurium and *S. flexneri* [27,30,48,49]. In line with this, exposure to bile salts also resulted in an increase in biofilm formation in *A. butzleri*. In addition, in the presence of 0.2% bile salts, a restructuring of the biofilm phenotype could be observed, changing from a predominantly superficial biofilm to a floating biofilm, where a pellicle at the air-medium interface is formed and can be correlated with an increase in bacterial cellular hydrophobicity. This pellicle, usually formed at the liquid–air interface of standing cultures has already been described for several bacteria and conditions [50].

Regarding the virulence, the relative expression of *cadF*, *ciaB*, *cheV*, *flaA*, and *luxS* genes of *A. butzleri* was evaluated, for Ab_2811 strain growing in the presence or absence of bile salts. Despite the association of *cheV* with chemotaxis activity and its role in different virulence mechanisms, such as biofilm formation and bacterial motility [23,51], the expression of this gene has not been changed following exposure of the bacteria to bile salts. This contrasts to the downregulation of the expression of the chemotaxis protein CheV found in *C. jejuni* in the presence of ox-bile [37]. In fact, both *flaA* and *cheV* gene expression have shown to be regulated by bile or bile salts in *Campylobacter* spp., which can influence the biofilm formation and bacterial motility [16,29,37,46]. The *luxS* gene encodes a protein that is responsible for production of autoinducers-2 that are important molecular communication signals between bacteria, for quorum sensing mechanism, and can be involved in bacterial motility and ability to form biofilm [52,53]. Nonetheless, the bacterial response to a *luxS*-deficient mutant may enhance the biofilm formation [54], or inhibit biofilm formation and decrease the bacterial motility, as described in *C. jejuni* [52,55]. In *Salmonella* spp. the presence of bile led to an increase in *luxS* gene expression associated with an increase in biofilm formation [53]. Although no significant difference was found, a trend towards increased expression of *luxS* gene in the presence of 0.2% bile salts compared with the control was observed in this work.

In the small intestine, bile salts are known to act as a signal to initiate microbial colonization [24]. Thus, the effect of previously exposed bacteria to bile salts was evaluated regarding its capacity to adhere to and/or invade into Caco-2 cells, since these are two steps required for successful colonization. Nevertheless, the results have shown no significant difference between bacteria exposed or not to 0.2% of bile salts, which is according with the study performed in *C. jejuni*, where the presence of the 0.1% bile acid deoxycholate did not affect the ability of the bacteria to adhere to INT-407 (human embryonic intestinal cells) epithelial cells [25]. Furthermore, when considering the expression of *ciaB* and *cadF* genes, which are homologues of the *Campylobacter* invasion antigen gene B and fibronectin-binding protein in *C. jejuni*, respectively, our results showed no influence of 0.2% of bile salts in the expression of these virulence genes. This finding is not in agreement with the study carried out in *C. jejuni*, where an increase in the expression of CiaB proteins was detected, when the bacteria were exposed to 0.05% sodium deoxycholate [29].

More studies will be needed in the future to elucidate the relationship between exposure to bile and the virulence mechanisms of *A. butzleri*. Accordingly, a transcriptomic or proteomic assessment of bile salt exposure at various time points could help to understand the impact of bile on the virulence features of *A. butzleri*. The use of these techniques has allowed for the evaluation of the effect of bile salts in the regulation of different cellular processes for several enteropathogens. For example, in *Campylobacter* spp., the presence of bile salts induced the expression of peritrichous pilus-like appendages, resulting in a highly aggregative phenotype [56,57]. Furthermore, in *C. jejuni* it was described that ox-bile can modulate the expression of proteins involved in chaperonin general stress responses, chemotaxis and motility, degradation of carbon compounds, protein translation and modification, and proton motive force ATP [37]. In *V. cholerae*, bile salts were shown to affect several virulence factors, such as increased motility due to increased expression of the flagellar genes, contrasting to a decreased production of the cholera toxin and toxin-coregulated pilus [58]. In *Wolinella succinogenes*, several strategies developed to tolerate bile were identified, namely several proteins involved in metabolic pathways that were associated with the amino acid metabolism and energy metabolism, providing protection to the bacteria under stress conditions [59]. Moreover, as the *in vitro* assays used do not represent the complexity found in the human gut, in the future, further studies that include other *in vivo* signals combined with bile should allow for deepening the response and survival of *A. butzleri* to the complex gastrointestinal tract.

## 4. Materials and Methods

### 4.1. Effect of Bile Salts on the Growth, Aggregation and Motility of Aliarcobacter butzleri

The strain used in this study was *Aliarcobacter butzleri* Ab_2811, which was isolated from the neck skin of a poultry carcass, in Portugal [60]. This strain was selected based on its use on previous studies [42,60], the available genome (Genbank accession number GCA_902500545.1), and being a food isolate it better mimics the infection transmitted to humans by the consumption of contaminated food. The strain was stored at −80 °C in Brain Heart Infusion (BHI, Liofilchem, Roseto d. Abruzzi, Italy) containing 20% (*v*/*v*) of glycerol (Labchem, Santo Antão do Tojal, Portugal). The strains were routinely cultured in Tryptic Soy Agar (TSA) (VWR, Leuven, Belgium) medium and incubated at 37 °C for 24 h, in microaerobic conditions (6% oxygen (O_2_), +/− 7.1% carbon dioxide (CO_2_) and 3.6% hydrogen (H_2_)). Before experiments, *A. butzleri* Ab_2811 was inoculated in TSA for 24 h at 37 °C under microaerobic conditions. Then, an overnight culture in 10 mL of Tryptic Soy Broth (TSB) (VWR, Leuven, Belgium) was prepared and incubated overnight, for approximately 16 h, at 37 °C and 100 rotations per minute (rpm), under microaerobic conditions. For all experiments, a negative control was performed consisting of TSB medium only, while the growth of the strain under study in TSB medium was considered the positive control. Bile salts (Sigma Aldrich, St. Louis, MO, USA), consisting of approximately of 1:1 mixture of sodium cholate and sodium deoxycholate, were used in this study.

### 4.2. Growth Curves

The growth of the strain in the presence of bile salts was performed according to the protocol previously described by Nickerson et al. (2017), with some modifications [27]. An overnight culture of *A. butzleri* was diluted 1:50 in TSB medium in the absence or in the presence of different concentrations of bile salts (0.2%, 0.5%, 1%, 2% and 5%). The cultures were grown with shaking at 250 rpm, 37 °C and under microaerobic conditions in glass tubes. Every two hours (0, 2, 4, 6, 8, 10, 12 and 24 h), aliquots were taken for viable counts (CFU/mL). All experiments were performed at least three independent times.

### 4.3. Hydrophobicity Test

The hydrophobicity of *A. butzleri* in the presence or absence of bile salts was measured by a salting-out method as described by Misawa and Blaser (2000), with some modifications [61]. After overnight culture, the strain was transferred to a new culture in glass tubes without or with 0.2% bile salts and the tubes were incubated for 6 h at 37 °C with shaking at 250 rpm, under microaerobic conditions. Subsequently, the incubated strains were resuspended in 2 mM sodium phosphate (Carlo Erba, Val-de-Reuil, France) at an optical density at 620 nm (OD_620nm_) of 1. Successive dilutions of ammonium sulfate (Labkem, Zelienople, PA, USA) at 4 M were carried out in 2 mM sodium phosphate in 96-well U-bottom plates (Thermo Scientific, Waltham, MA, USA). A volume of 25 μL of bacterial suspension was added to each well to a final volume of 50 μL. The plates were incubated at 25 °C (room temperature) overnight. After incubation, the minimum concentration of ammonium sulphate forming bacterial aggregation was determined by evaluation at naked eye, and the value was used for the hydrophobicity index. Hydrophobicity is inversely correlated with ammonium sulfate concentration causing bacterial aggregation. This test was performed at least three independent times.

### 4.4. Motility Assay

*A. butzleri’s* motility was evaluated as previously described by our research team [62]. Plates of TSB with 0.4% of agar (GRiSP, Porto, Portugal), with and without supplementation with 0.2% of bile salts were stabbed in the center with 5 μL of a bacterial suspension adjusted to 10^8^ CFU/mL from an overnight culture. The halo of motility was measured at 24 h and 48 h after incubation at 37 °C in microaerobic conditions. This experiment was performed at least three independent times.

### 4.5. Biofilm Formation and Quantification Assay

The ability of *A. butzleri* to form biofilm was evaluated in the presence or absence of bile salts. Briefly, an overnight culture was centrifuged and resuspended to an OD_620nm_ of 0.2 in TSB with or without 0.2% of bile salts. Then, 100 µL or 1 mL of bacterial suspension was transferred to a 96-well U-bottom plate (twenty wells) or sterile glass tubes (duplicates), respectively. TSB and TSB with 0.2% of bile salts were used as negative controls. After that, the 96-well U-bottom plate was incubated for 2 days, and the glass tubes incubated for 2, 5, and 7 days at 37 °C under microaerobic conditions. Biofilm formation was quantified using the crystal violet assay, following the protocol described by our research team [42]. After the medium was removed, the plate and tubes were incubated for 1 h at 55 °C. Subsequently, the biofilm was stained with crystal violet (Amresco, Solon, OH, USA) at 0.1% (*w*/*v*) in deionized water and incubated for 15 min at room temperature, followed by washing three times with distilled water and incubating again at 55 °C for 15 min. After drying, the biofilm was de-staining with a 30% (*v*/*v*) methanol (Fisher Chemical, Loughborough, UK) and 10% (*v*/*v*) acetic acid (Fisher Chemical, Loughborough, UK) solution. Then, 100 µL and 1 mL were transferred to a new plate of 96-well plate and 24-well plate (VWR, Radnor, PA, USA) (in the case of the tubes), respectively, and the absorbance at 570 nm was determined. The assay was performed at least three independent times.

### 4.6. Confocal Laser-Scanning Microscopy Imaging of Biofilms

The images of the biofilm of *A. butzleri* were acquired by the protocol previously described by Wang et al. (2020), with some modifications [63]. After overnight culture, a bacterial suspension with an OD_620nm_ of 0.2 in a final volume of 10 mL TSB without or with 0.2% of bile salts was prepared in 50 mL conical tubes. Then, a sterile glass coverslip has been vertically added to the culture and subsequently incubated statically at 37 °C under microaerobic conditions for 5 days. After incubation, the glass coverslip with the biofilms was carefully removed with forceps and placed in sterile petri dishes, washed with ultra-pure water and stained with 200 μL of 0.01 mM SYTO 9 solution (Invitrogen, Molecular Probes, Eugene, OR, USA) and incubated for 30 min in the dark at room temperature. Finally, the glass coverslip was washed to remove excess of fluorochrome. Confocal microscopy images were obtained on a LSM 710 AxioObserver microscope using a 40× objective (Zeiss, Jena, Germany). The confocal microscope settings of excitation and emission of SYTO 9 were 488 and 535 nm, respectively. The stack images were obtained by scanning the biofilms along the Z-axis at 0.5 µm intervals. Three-dimensional (3D) reconstructed images were generated using Zen (3.5 blue edition) software. To quantify the thickness of the biofilms, confocal images were analyzed using COMSTAT software [64]. The assay was performed at least three independent times.

### 4.7. Relative Expression of Several Virulence Genes

The overnight culture was diluted to a new culture without or with 0.2% of bile salts and incubated at 37 °C with shaking at 250 rpm under microaerobic conditions in glass tubes. Samples were collected for total RNA extraction at the middle of the exponential phase (approximately 6 h). The aliquot was centrifuged, and the cells were washed twice, and total RNA was isolated using TripleXtractor reagent (GRiSP, Porto, Portugal) following the manufacturers’ instructions. After, DNase I (Thermo Scientific, Vilnius, Lithuania) treatment was performed, followed by cDNA synthesis. For each sample, 1 μg of total RNA was reverse transcribed using the Xpert cDNA Synthesis Mastermix kit (GRiSP, Porto, Portugal). RT-qPCR was performed with specific primers for the *cadF*, *ciaB*, *cheV*, *flaA*, *luxS* and *16S rRNA* genes (Table 1). For a final volume of 10 μL, qPCR mixture was constituted by 5 μL of NZY qPCR green master mix (2×) (NZYTech Ltd., Lisbon, Portugal), 0.4 μM of specific primers and 1 μL of cDNA. The program used had three steps: first, 2 min at 95 °C, followed by 40 cycles of 5 s at 95 °C and 30 s at 60 °C, and finally 5 s in increasing gradient to 95 °C, in a CFX Real-Time PCR System (Bio-Rad, Hercules, CA, USA). For analysis of relative expression, the comparative threshold cycle (2^−∆∆CT^) method was applied, *16S rRNA* was used for gene normalization [65].

### 4.8. Adhesion and Invasion Assays to Caco-2 Intestinal Epithelial Cells

The ability of *A. butzleri* to adhere and invade host cells was analyzed in Caco-2 cells, an epithelial cell line of human colorectal adenocarcinoma, based on the protocol previously described by our research team [42]. Caco-2 cells were grown in tissue culture flasks with Dulbecco’s modified Eagle medium (DMEM, Sigma-Aldrich, United States of America) supplemented with 10% fetal bovine serum (FBS, PAN-Biotech, Aidenbach, Germany), 1% non-essential amino acids (Lonza, Basel, Switzerland) and 100 µg/mL streptomycin and 100 U/mL penicillin solution (Sigma-Aldrich, St. Louis, MI, USA), and incubated at 37 °C in atmospheric of 5% CO_2_. For the assays, 24-well plates were seeded with 1 × 10^5^ cells/well of Caco-2 and incubated for 48 h in the same conditions. Bacterial suspensions were prepared after incubation for 6 h without or with 0.2% of bile salts. In the adhesion assay, the Caco-2 cells were infected with a multiplicity of infection (MOI) of about 100 for 3 h at 37 °C in a 5% CO_2_ atmosphere. After incubation, cells were washed three times with Phosphate-buffered saline (PBS, Lonza, Walkersville, ML, USA) and Caco-2 cells lysed with 1% Triton X-100 (VWR, Solon, OH, USA). For the invasion assay, a gentamicin protection assay was performed, where extracellular bacteria were killed by incubation with 125 μg/mL gentamicin (Sigma-Aldrich, St. Louis, MI, USA) for 1 h, after 3 h of infection. Then, the cells were washed to remove residual antibiotics and lysed with 1% Triton X-100. The adherent and invading bacteria were determined by successively diluting the lysates and plating on TSA plates by the drop-plate method. Each assay was performed at least three independent times, in triplicate.

### 4.9. Statistical Analysis

Data were presented with mean values ± standard deviation (SD) or standard error of the mean (SEM) according to the experiment. The statistical analysis of the data was analyzed with software GraphPad Prism (GraphPad Software version 8, USA), using the two-tailed Student’s *t*-test. Differences between the mean values to *p* < 0.05 were considered statistically significant.

## 5. Conclusions

As an adaptation mechanism to environmental/external stressors, *A. butzleri* should be able to induce changes in its physiological response to promote survival and proliferation in the host. This investigation provided for the first time information about the survival ability of *A. butzleri* in relation to bile salts, an enterohepatic stress factor, as well as changes in several virulence mechanisms. *A. butzleri* was able to survive bile salts at the physiological concentration found in the human gastrointestinal tract. In addition, exposure to bile salts has been found to modulate several bacterial virulence factors, such as bacterial motility, the ability to form biofilms and biofilm phenotype. Furthermore, a modification in bacterial hydrophobicity and an increase in the expression of the *flaA* gene in the presence of bile salts were observed. These findings suggest possible routes of adaptation of *A. butzleri* to this specific niche in the host environment and opens doors for new studies to be carried out on the influence of bile salts on the pathogenicity of *A. butzleri*.

## Figures and Tables

**Table 1:** Oligonucleotide sequences used in RT-qPCR.

Primers	Target Gene	Sequence	Reference
cadF_F	*cadF*	5′-CTCCAGTTGCTGCACCAAAA-3′	This study
cadF_R		5′-CCAATATTGTCAACTTTTGCACC-3′	
ciaB_F	*ciaB*	5′-TTGGCAAACTTCATGGACTGC-3′	This study
ciaB_R		5′-AGCAGTAATTCCTCCATGTCCT-3′	
cheV_F	*cheV*	5′-TGAAGAGGTGGCGATAAATG-3′	This study
cheV_R		5′-GCTTTTAATCCAAGCCAAGC-3′	
flaA_F	*flaA*	5′-AGTTGCACCAGCTGACATTT-3′	[66]
flaA_R		5′-AGTTGGTGAAGGAAGTTCCGA-3′	
luxS_F	*luxS*	5′-GAGCACCTTTTTGCTGGATT-3′	This study
luxS_R		5′-TTCCAAGCAACTGCAACTTC-3′	
P338_F	*16S rRNA*	5′-ACTCCTACGGGAGGCAGCAG-3′	[67]
P518_R		5′-ATTACCGCGGCTGCTGG-3′	

## Data Availability

Data is contained within the text.
